# Clinical experience with clozapine in patients with severe intellectual disability and behavioral disorders.

**DOI:** 10.1192/j.eurpsy.2023.367

**Published:** 2023-07-19

**Authors:** N. Laherrán Cantera, R. Palacios-Garrán, L. Jiménez Suarez, C. Rodriguez Martín, J. Machuca Sicilia

**Affiliations:** Mental Health Unit, Jerez University Hospital, Jerez de la Frontera, Spain

## Abstract

**Introduction:**

It is estimated that the prevalence of severe Intellectual Disability (ID) is 6 per 1,000 people. ID is sometimes the cause of Behavioral Disorders (BD) with aggressive and impulsive behaviors that make family and social life difficult. However, despite its high prevalence, the number of studies on it is very scarce.

When BD appears, it should be evaluated if there is a physical or psychiatric cause that causes it and assess non-pharmacological treatments. If they are insufficient, treatments such as risperidone are used to manage BD. When these are ineffective, the use of drugs with greater difficulties in their effects and clinical management, such as clozapine, is required.

**Objectives:**

The objective is to describe the use of clozapine in patients with severe ID associated with BD.

**Methods:**

Retrospective descriptive study. Patients older than 18 years with severe ID and BD, treated with clozapine for at least two years were included. Those with medical or psychiatric comorbidity were excluded.

**Results:**

The sample consisted of 12 patients, 16.67% women (n=2) and 83.33% men (n=10), aged 47.57±9.27 years. Prior to the introduction of clozapine, a mean of 2.67±1.21 antipsychotics had been tested. The mean dose of clozapine was 264.24±70.50 mg/day. The patients had received treatment for 51.57±25.67 months, following the usual controls. None had hematological adverse effects or other serious adverse effects.

**Conclusions:**

Clozapine can be an effective and safe therapeutic alternative in the treatment of BD in intellectual disabled patients which do not respond to other treatments. The clinical benefits of clozapine treatment seem to outweigh the potential risks associated with the treatment. However, more studies are needed to evaluate the effects of clozapine in patients with intellectual disabilities.

**Disclosure of Interest:**

None Declared

